# An IoT-Based Ship Berthing Method Using a Set of Ultrasonic Sensors †

**DOI:** 10.3390/s19235181

**Published:** 2019-11-26

**Authors:** Ahmadhon Kamolov, Suhyun Park

**Affiliations:** 1 Department of Computer Engineering, Dongseo University, 47 Jurye-ro, Sasang-gu, Busan 47011, Korea; ahmadxonk@gmail.com; 2 Division of Information and Communication Engineering, Dongseo University, 47 Jurye-ro, Sasang-gu, Busan 47011, Korea

**Keywords:** smart ship berthing, smart ships, smart ports, internet of things (IoT), marine IT

## Abstract

It is indisputable that a great deal of brand new technologies such as the internet of things, (IoT) big data, and cloud computing are conquering every aspect of our life. So, in the branch of marine technology, the mentioned technologies are also being applied to obtain more features and to automate marine-related operations as well as creating novel smart devices. As a result of this, traditional ports and ships are being replaced by smart ports and vessels. To achieve this transition, numerous applications need to be developed to make them smart. The purpose of this paper is to present a dedicated an IoT-based system for automating linkage procedures by searching for available locations via port-mounted sensors and planned ship notification. In the experimental system, we have used smartphone as an alternative to the client-side vessel of the system and created an Android app called “Smart Ship Berthing” instead of the charging program, for instance, NORIVIS 4, VDASH, ODYSSEY, etc. To test our proposed server-side system, we used Raspberry Pi with a combination of an ultrasonic sensor to detect the ship and modify the empty berth for anchoring. The experimental results show that the set of UR sensors have high accuracy to detect ships at the port for ship berthing and our proposed system is very amenable to implementation in the real marine environment.

## 1. Introduction

Embedded technologies such as big data, artificial intelligence and the internet of things, represent the adoption of Industry 4.0 technology in the maritime field. A variety of new communications technologies will dominate the next generation of communications between smart ships and smart ports, including satellite communications. It is no paradox that humans are surrounded by technologies and that is why intelligent technologies do almost all the work we do during our daily existence. Therefore, humans are trying hard to develop numerous automation areas, for instance, smart strollers, smart cars, smart watches, smart cameras, and smart umbrellas, etc. [1]. Even the places where we study [2], live, and work have become smart. In this context, all objects and elements are replaced by their smart alternatives. For instance, traditionally modeled ships and ports are evolving into smart ships and ports and this offers several premium opportunities for research in marine science. It should be mentioned that large companies from developed countries are still studying these two big projects. Thus, enormous thriving corporations in Korea, Japan, and China are proposing IoT solutions for smart ships [3,4,5,6] and developed countries in Europe (such as Germany and The Netherlands) are actively working on smart ports. In this study, we suggest a system that supports berthing by automatically locating the berth in a port based on the IoT. Upon receiving a request from a ship arriving at the port, the system automatically sends data about the location where it could dock before reaching that location. The system uses information from a range of ultrasonic sensors associated with the available space. However, everything could be done by automating ships. The new concept of smart ships focuses not only on collecting and controlling data independently, but also refers to free communication with other ports and ships. In this matter, the role of different sensors, devices, and software installed in ports and smart ships is important when performing these functions. In particular, the demand for software for smart devices is expected to increase significantly over the next few years. Each transfer requires a special automation system. In order to fully automate and personalize the entire ship and port system, the parts and process must be automatically split before the goal is achieved. At this point, we can conclude that with such a large object, it is part of the initial process automatically and the entire system turns into an intelligent or automatic monitoring system. When newly emerging technologies such as the IoT are applied to actual transportation procedures, one of the available means of transport is the port berthing process. The system currently in use has many drawbacks. In this study, we suggest a system that supports berthing by automatically locating one in the port based on the IoT. The system uses information from a range of ultrasonic sensors associated with the available vacant berths.

After going through the introduction of the system in Section 1, the rest of the paper includes the following sections: Section 2 is about the background and related works, and also gives information about the IoT in detail and brief information about smart ships, smart ports and also the role of the most recent model technologies in marine technology. Section 3 illustrates the problem of ship berthing, Section 4 describes the proposed system architecture, design, and working scenario, Section 5 is devoted to the implementation and experiments, whereas the experimental results appear in Section 6. Section 7 is for the discussion and finally, the conclusions of this work are presented in Section 8.

## 2. Related Works

### 2.1. Internet of Things—IoT

The internet of things (IoT) is also called the internet of objects is a means for making connections between things, sensors, actuators, and other smart devices to facilitate human-to-object and object-to-object communications [7,8,9]. The IoT is a network of physical devices, communications, machines, and other items installed with electronics, software, sensors, actuators, and network connectivity which allow objects to gather and exchange the data [10,11]. Each item is unequivocally identifiable via its fixed computing system, however, can interoperate within the existing internet infrastructure [12]. Experts expect that by 2020 the IoT will comprise approximately 30 billion items [13,14,15,16,17]. Having collected all the definitions about IoT we can define our version as follows: IoT offers the capability of fixing sensors, actuators and software to any object and thing surrounding us that we can touch, or see in our life and use, monitor and operating the data existing in those objects in any situation, anytime and in any area. 

#### 2.1.1. Main Architectures of the IoT

There are four main types of IoT architecture: three-layer architecture, middleware-based architecture, service-oriented architecture (SOA) architecture, and five-layer architecture. Among these four sorts of architecture, the most typical one is the three-layer architecture. For this reason, we decided to focus on this architecture rather than the others. As suggested by its name, the three-layer architecture consists of three simple layers [18,19], which are as follows:

- Application Layer
- Network Layer
- Sensor Layer

##### Application Layer

The network layer sends the data to this layer and receives the data. Then the data is utilized to provide the demanded operations or services. For instance, the application layer is able to provide a storage service to back up accepted data into a database or supply an analysis service to assess the received data for prognosticating the forthcoming state of physical devices. Several applications fall in this layer, for instance, smart homes, smart wearables, smart grids, smart transportation, smart cities and others can be classical examples of this [19,20,21,22,23].

##### Transmission Layer

The transmission layer or the network layer is used to obtain the data processed by the sensor layer and to define ways to transfer information and data to the IoT hub, devices, and applications through integrated networks. The network layer is the most important layer in the IoT architecture, the reason being that a variety of devices (switches, hubs, cloud computing performance, gateways etc.), and different communication technologies (ZigBee, Bluetooth, LTE, 5G, 6LoWPAN, Wi-Fi etc.) are combined in this layer. The network layer must provide data to or from different objects or applications, through gateways or interfaces between heterogeneous networks, and use different communication technologies as well as protocols.

##### Sensor Layer

**Figure 1:**
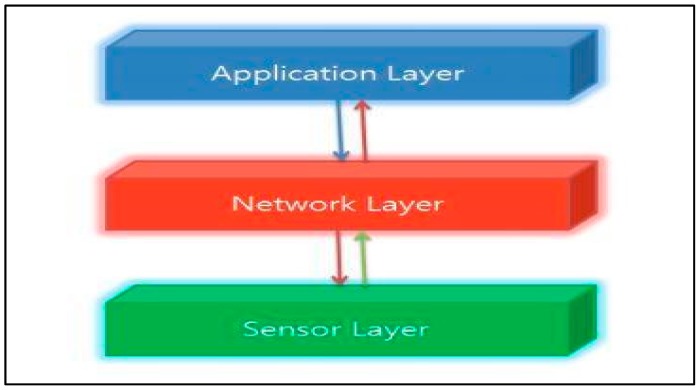
Three-layer IoT architecture.

The sensor layer, also referred to as the perception layer provides the connections with physical items and components by means of actuators, sensors and other sensing technologies such as RFID, WSN, GPS, etc. The main gist of it is that the sensor layer provides the physical and digital world to get in touch with each other. Its prime goals are to unite the things in the IoT network and to measure, gather, and process the main data linked with these items by means of ready smart objects, transferring the processed data to the upper layer through the layer interfaces [19]. See Figure 1 below.

#### 2.1.2. IoT Elements and Technologies

To comprehend the IoT and IoT architecture, in order to know deeply the ways of connecting IoT parts with each other and the way as well as the means for exchanging data, initially, we should learn more about IoT functionality [23]. Here, we have discuss IoT functionality and define six tasks, namely identification, sensing (feeling things), communication technologies, computation and management, IoT services and semantics which are essential to run the IoT and their various methods as well as their contents.

- Identification

As we indicated in previous sections the basic definition of IoT is as an amenity to create a connection with any object at any time and in any place. After making this connection we should be able to send an order or receive all the data existing in that object without any hardship. With this aim, it is quite significant to get in touch with the objects or devices which we want to connect. It is estimated that this significance will increase in forthcoming years when the network consists of billions of devices. There are several identification methods that can achieve this task. For instance, electronic product codes (EPCs) and ubiquitous codes (uCodes) are stated patterns of these methods [23,24], and also other identification technologies [25]. Apart from this, addressing has also a vital role for informing the objects that we desire to make a connection. Simply put, in order to connect with an object in the network correctly, we need both an ID and an IP. The ID identifies the device itself and the IP provides the address of that device in the network. IPv4, IPv6, and 6LoWPAN are some of the addressing methods used [23,24,26,27].

- Feeling Things

Feeling things is to comprehend the working process of sensors. It means that feeling a thing involves collecting all the data that exists via certain sensors. To explain this concept more clearly, we can state, for example, that to check the heat and humidity, an object (room) will use temperature and humidity sensors. Or, in another example, in order to get information about the color of a device, a color sensor or a camera can possibly be utilized. Generally, sensors are categorized into two sorts: active and passive. The paramount difference between them is that active sensors emit energy and the energy obtains information by evaluating the reaction of external objects to this energy, while passive sensors emit no energy, and in stark contrast to active sensors, they gather information via accepting the energy of the objects around them [28].

- Communication Technologies

All objects and the sensors which are linked to objects and the technologies that can connect available devices to each other as well as to the network can be grouped within the communication technologies of the IoT. Some of these technologies are Wi-Fi, Bluetooth, NFC, RFID, ZigBee, Z-Wave, LTE, LTE-A, WiMAX and all 3G, 4G, 5G technologies. By means of these technologies objects acquire the ability to communicate with each other and they can easily exchange any kind of data in anytime and anyplace taking into consideration the variety of IoT devices that are able to communicate, considering their aim and the measurement technique employed. Examples for particular types of sensors are humidity sensors that detect humidity (as the amount of water vapor) in the air or a mass hygrometer soil moisture sensor, light sensors that detect the presence of light (visible or invisible) like infrared sensors, humistors, photodetectors, and flame detectors, temperature sensors that measure the amount of heat or cold that is present in a system like thermometers, calorimeters, temperature gauges, biosensors that detect various biological elements such as organisms, tissues, cells, enzymes, antibodies, and nucleic acids, for example, blood glucose biosensors, pulse oximeters, scintillators and electrocardiographs, radiation detectors like Geiger–Müller counters or neutron detectors detect radiation in the environment. All these devices then exchange information mutually through diverse communication protocols, so when choosing communication technologies for IoT systems, commonly the features of those individual technologies and working principles of the target system are considered the main target. For instance, in order to exchange data in a minimum distance communication—NFC, ZigBee, Bluetooth—for a normal distance communication WiFi, and for a long or maximum distance data exchange methids such as LTE, LTE-A, WiMAX, LoRaWan(LoRa) and such communication technologies can be helpful and suitable for exchanging data with other devices. In [29,30,31] in depth information about communication technologies is presented.

- Computation and Management

This is one of the critical parts of creating a system based on IoT because this block receives data collected by identified sensors and devices via diverse sorts of communication technologies and it performs the calculations and commands in the system. Briefly we can describe it as the hardware and software for IoT applications. Arduino, Raspberry Pi, Intel Galileo, and smartphones are examples of widely used hardware devices and Linux, and Android are the most utilized software [32,33,34,35,36].

- IoT Services

There are four kinds of IoT services [23,37,38,39]. One of them is “identity-related services” that are mostly utilized to place real-world objects to the virtual world by identifying them. Another one is information aggregation services. They gather and sum up raw sensor measurements that have to be processed and reported to the IoT application. The third one is awareness services used to sort and summarize the information taken from information aggregation services. Lastly model ubiquitous services collaborative aware services focus on creating a opportunity to be used by any human who needs them anytime.

- Semantics

According to the working principles of IoT architecture, a huge amount of information and messy data enter the network of sensors and other smart devices. Sorting out and getting the target is a complicated issue if we utilize current network technologies. For this reason the IoT directs the attention towards applying semantic technologies in the network. The initial point for creating a beneficial set of actions is usually interoperability and semantically describing the data and in this context, creating actionable information is the main objective.

### 2.2. IoT, Cloud, BigData in the Marine

The utilization of IoT, cloud, and big data under the water and over the water has nearly the same features with the objects and industries on land. The differences related with this issue are mostly being addressed on vessels and in ports. for this reason, considering the iot, Cloud, and Big Data currently two terms are becoming popular in this industry. These terms are surely smart ships and smart ports. IoT, cloud, and big data solutions in the marine sector state that the main two objects, ships, and ports, of the industry, are equipped with a number of devices and sensors and those devices are connected with each other. As a result, they can exchange information mutually and with other external objects. In a nutshell, according to the conception of the forthcoming smart ship, each ship can communicate with other ships, ports and some of the systems in their external world.

#### 2.2.1. Smart Ships

As we mentioned above, like in other industries, the shipbuilding industry is also becoming enhanced by modern technologies like IoT, cloud, and big data. Like other industries, in this industry scientists are trying to minimize human labor through utilizing robots, diverse machines and smart systems. It is because the appearance of the term “smart ship” in the hi-tech world and in several research works did not happen accidentally. If the giant companies which make technological devices start to manufacture vessels provided and equipped with various sensors, fixtures and software which allow the whole ship to work as a system, in that case, we can really call such ships smart. This means, briefly stated, that we define these smart ships which we are trying to build as floatable robots in the sea because like robots they can be operated by means of computer systems and their motions can be totally controlled. For more information we can highlight the “iDolphin”, produced in China, a ship with the features as we have emphasized that was revealed for the first time at the Smart Ship Development Forum & Smart Ship Demo andp named “Great Smart” [40,41,42,43]. In order to obtain the title of smart, ships are expected to have the following specifications and functions.

(A) Various sensors and smart devices needed

If we consider ships as islands floating in the sea and consider that this floating island contains humans, at that point we can view ships as small cities where people dwell. From this point of view, we can summarize that in comparison with cities, ships also have the necessity of obtaining diverse kinds of information, so future ships need to be equipped with a number of sensors. Among them, sensors such as motion, humidity, temperature, position sensors, light sensors, acceleration sensors and others which are being utilized in various IoT projects can be genuine examples. Such sensors which gather data from the objects attached to them and from the world around them are generally considered the main technical part of monitoring the ship condition via diverse software. For instance, humidity and temperature sensors are vital to control the climate of interior of the ship and position, and acceleration sensors are beneficial for observing and monitoring the location, movement trajectory and the speed of the ship.

(B) Software needed

As in all IoT projects that exist, smart ship-building requires different types of software. The software provides diverse functions which are applicable to collect data from the sensors that we highlighted above. This means employing certain methods for the different sensors that are used to accomplish a certain task is required in creating software.

(C) Various communication technologies needed

If a ship contains several sensors and the software which gathers information through the sensors, having this feature alone is not enough to call it “smart” because, as we highlighted above, in order to consider a ship a smart version it must exchange integral data with other ships as well as ports. For this purpose, the ship should be equipped with various types of means of communication. 4G, 5G, LTE (LTE-M), Wi-Fi, WiMAX are the examples of these technologies.

#### 2.2.2. Smart Ports

**Figure 2:**
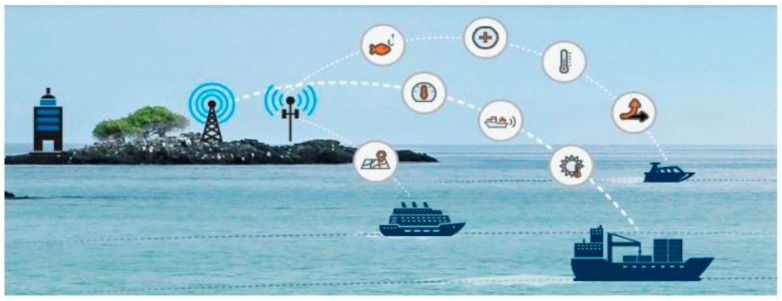
Communications between ships and ports.

Smart ports are also considered as a novel concept like smart ships and smart cities. Its requirements are nearly identical to those smart ships and smart cities. Only according to the type of the port several additional services can be included when building a smart port. As a topic regarding the types of the ports that requires extensive clarification, we only focus on smart port building for traditional ports. Thus smart ports aim to include various technologies and amenities. Each berthing place at the port should contain automatically operated devices and sensors, moreover, the software which is utilized in those devices and other extensive systems should be included. Besides this, smart ports are equipped with various communication technologies to exchange data with other ports, smart ships and with smart cities as well. The services created in smart ports are composed of automatic monitoring and controlling the location of the ships at the port, the movement schedules of the ships, loads, and the ride of the passengers and so on. The communication between smart ships and smart ports is depicted in Figure 2 [43].

#### 2.2.3. Big Data and Cloud in Shipping

Modifying ships and ports into smart versions is another problem and the drawbacks are connected with the vast amount of data collected, processed and exchanged. It is obvious that these two objects are really huge are the point of measurement. Concerning this, involve not only hundreds of sensors but the number of sensors are likely to even be several thousand. The amount of data that is transferred by the sensors in real time is really vast. Big data, cloud computing, and such technologies are attractive to solve the resulting problems in the field as they are so effective at working with big and real-time data.

### 2.3. Related Works

**Figure 3:**
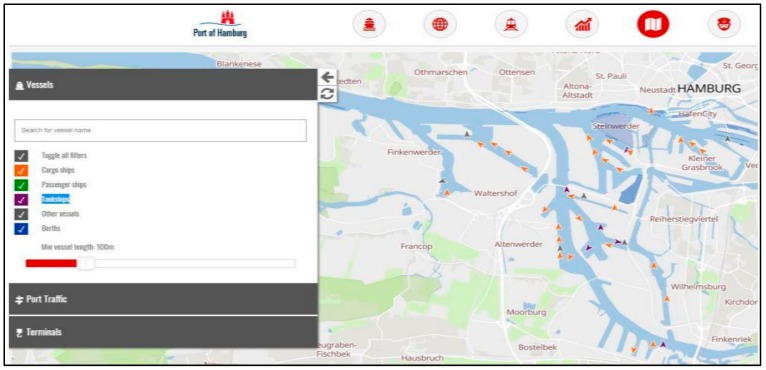
Hamburg port interface.

If we want to talk about topics and research that can be related to smart ships and smart ports, there are numerous studies in this area. Based on their functionality, the smart ship concept is quite similar to the smart car concept because cars and ships are both mediums of transport and their job is the movement people and their stuff. Becoming a smart thing involves adding hardware, software, and sensors to things in order to enhance their functionality and make them work automatically. That is why we can say the smart ship and smart car concepts are the same based on their functionality. In this case, Google’s or Uber’s unmanned or should we say smart cars can be an example of related research because in all of them, in order for the whole system to work as planned, every part of those systems should work perfectly. What we mean that those systems are divided into parts to do some particular jobs. For example, knowing the weather conditions of someplace by measuring humidity and temperature using particular sensors like the DHT22 or checking location by GPS to track an object are a common independent task of different big projects like smart carts, smart ships, smart ports and smart cities. If we want to talk about projects that are really related to our work, we can say that there is no such 100% similar project yet, but of course, some of the big ports around the world are already presenting interactive services to their clients by using the latest technologies. The port of Hamburg in Germany can be a pattern for this. One of the interactive services of this port for their clients is their clients can search vessels and places or check their condition in real-time on the port’s website [44]. This is illustrated in Figure 3 where colored pointers are ships and their color represents their type.

**Figure 4:**
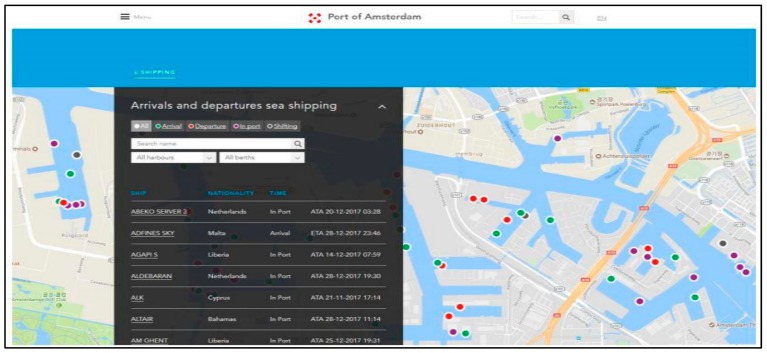
Amsterdam port interface.

Another port, Amsterdam, has launched multiple apps. The “I am Port” app offers real-time information on ships’ location and itineraries at the port. In addition, we can find information about arrivals and departures, size, draft and berth of each ship at the port. They also mark ships with different colors but here color does not mean a type of ship, but rather it means a status of that ship. For example, greens are arrivals, reds are departures, as seen in Figure 4 [45].

According to [5], the maritime start-up company We4Sea is developing an application for ships that is able to cut fuel costs by up to 20 percent. The We4Sea system gathers some operational data from the ship like its position, speed, heading, and engine data and sent them to shore to be combined with other data like the weather condition of the sea, wave heights, currents, and wind. After combining those data, system algorithms and energy models translate them to actionable information to optimize the operation and configuration of the ship.

Oceanstar [6] is an intelligent berthing system that can help to berth vessels on the shore. The system shows the position and movement of the ship. It collects and measures vessel key data, and presents them in a simple and easy-to-read display. This system uses high-performance global navigation satellite system (GNSS) data to improve navigational safety and reduce operating costs.

Similar research and applications has been done in different fields which are similar to our system such as studies based on vehicle to vehicle (V2V) [46,47], vehicle to infrastructure (V2I) [48,49], and device to device (D2D) communications. For example, autonomous driving, road worker, construction zone warning, traffic information (traffic jam ahead warning systems and vehicle-to-grid communications). Because of the similarity of the working scenarios, smart car parking or car parking is essentially identical to our work.

In [50], an image processing facility-based smart parking system is proposed It obtains information about the available parking lots and gives it to cars. In [51] an IoT-based cloud-integrated smart parking system was presented that consists of an onsite deployment of an IoT module that is used to monitor and signal the state of availability of each single parking space. Reference [52] introduced a solution for finding high demand parking placea. The authors used ultrasonic sensors to detect vacant parking spots and transmit the data via a wireless sensor network using the ZigBee protocol. In [53,54], the authors designed a smart parking system which enables users to find the nearest parking area and gives the availability of parking slots in that respective parking area. Most authors utilize an ultrasonic sensor to detect empty space, which is similar to our work.

## 3. Problem Analysis in Ship Berthing

Ships are commonly considered a huge vehicle. An enormous number of hardware and software technologies are required to make them smart and undoubtedly, accomplishing those demands really needs a lot of time as it cannot be carried out within a short period of time. By considering small parts of a ship, we can create uncomplicated software gradually.

Through this research, we introduce our diminutive system that is fully applicable in the arriving and berthing port access process. We strongly expect that the proposed system will succeed in reaching ships while arriving at the port and help them find a place automatically, which has been experimentally proven. Before describing the system, it is necessary to understand the communication between ships and ports in order to comprehensively appreciate the amenities of our system.

In the shipping industry, not only is communication between ships practical but also communication between ships and ports is critical and the main proportion of this communication occurs when ships arrive at a port. Currently, ships waste too much time in berthing at the port as the task may last from two hours to several days. One reason for this problem is the presence of a crowd of ships in the port and the other trade-off is the huge amount of information exchanged between ships and ports. The data required by the port includes complete information regarding the name of the ship, the owner, the crew, the overall dimensions of the ship and its condition, as well as the expected time of arrival and departure of the ship. In these days such a vast amount of the data transfer procedure is managed with the help of portable radio transmitters. That is one of the main reasons so much time is wasted during berthing. Besides that, having exchanged the data, a responsible member of the port staff must arrange a place for the ship to moor and this procedure may cause trouble as well as inconvenience. As an example, we include in this paper a typical dialogue between a port and a ship that are exchanging data before the ship gets permission to berth. In the dialogue, we highlighted a circumstance where the captain of the ship called “TITANIC” and the person responsible for controlling the port system are having a conversation [42]. Appendix A shows this example of conversation between port and ship, illustrating how the berthing procedure adds additional time, problems and cost as well.

## 4. Proposed System

### 4.1. Architecture

**Figure 5:**
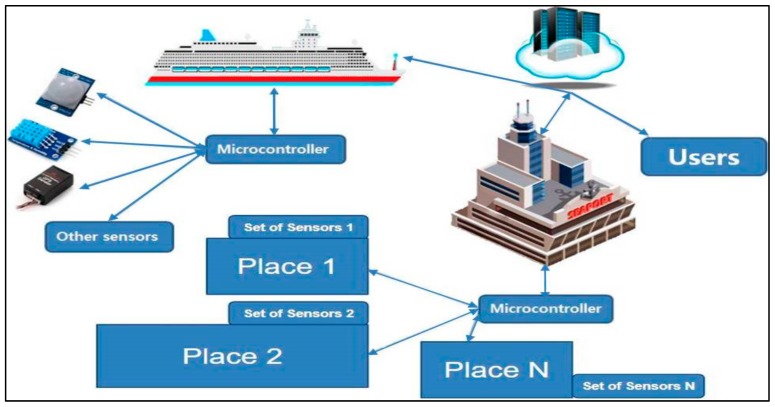
High-level architecture of the proposed smart berthing system for ships [37,55].

Nowadays the arrival and departure of ships at the port is getting very busy. As can be seen in the pattern given in the preceding section, ships arriving at a port often cannot find a place to berth easily and it is a really complicated situation. Our IoT-based smart berthing system can help vessels and ports in such situations. The principal purpose of the system is to help to berth vessels which have arrived at the port and help exchange data regarding their situation. The system is able to manage several tasks automatically like receiving all the data about available berths by means of a set of ultrasonic sensors affixed at the port and having obtained the data, it transfers it to the vessels looking for a place to berth. The system categorizes the online data received from the set of sensors and offers the ship an optimal place concerning location, quantity and other criteria. As many of the applications of IoT-based systems are created by means of three-layer architectures, our proposed system is also based on this architecture (see Figure 5).

#### 4.1.1. Smart Berthing System for Ships (Application Layer)

Using the Application layer ships send a request for a place to berth to the port. The application layer consists of the software which is suitable for transmitting and receiving the data from and to the port.

#### 4.1.2. Smart Berthing System for Ships (Network Layer)

This network layer provides a means for communications between the application layer and sensor layer that can be used in our system based on diverse technologies like Wi-Fi, WiMAX, LTE (LTE-A, LTE-M), etc.

#### 4.1.3. Sensor Layer of the Smart Berthing System for Ships

The sensor layer of the system can be viewed as common sensors that are fixed at the port. This set of sensors works to elucidate a certain place if there is a vessel at the port or not. Then, with the help of the network layer it transfers the information to the application layer, that is, it transmits data to the vessels. Ultrasonic sensors are a superlative sensor to specify the berth status owing to their affordable cost and convenience and that is the reason why we utilized a set of ultrasonic sensors in our system. As illustrated in Figure 5, all berths have their own set of sensors. Bigger sized berths, for example Place 2 have more sensors, while smaller ones like Place 1 have less sensors.

### 4.2. High-Level Design

**Figure 6:**
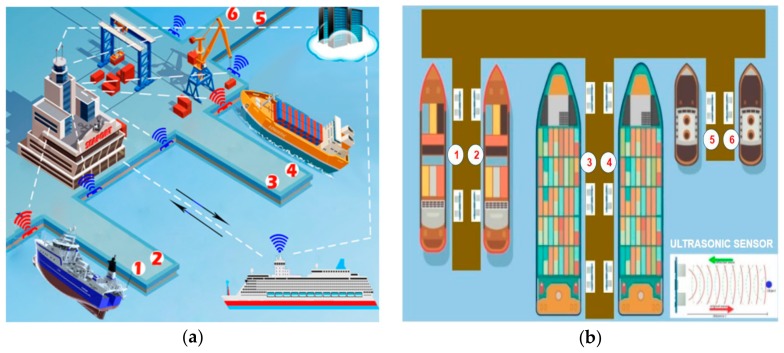
(**a**) The first version of the design for the proposed system; (**b**) the updated version of the design for the proposed system [37,55].

Furthermore, we set up high-level visualization for the proposed system as shown in Figure 6a,b. Figure 6a is the first proposed design we introduced in our previous study [55] and Figure 6b shows the upgraded design of the current proposed system. In the designs shown below, ships can be anchored at each berth in the port equipped with a particular set and number of sensors. Every space at the port has its own set of sensors and numbers. In Figure 6a, the red colored sensor means that the space is occupied and that the sensor provides “busy” information. The blue sensor indicates that the space is empty and that the sensor indicates “empty” information. The above scenario is the same as our first one and when a set of sensors are busy is means there is ships in the berth, and if not the place is empty.

### 4.3. Working Scenario

**Figure 7:**
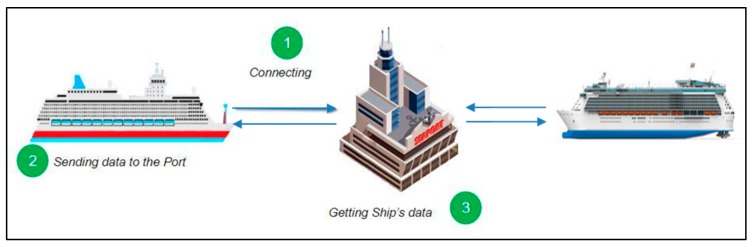
System scenario, steps 1 to 3 [37,55].

**Figure 8:**
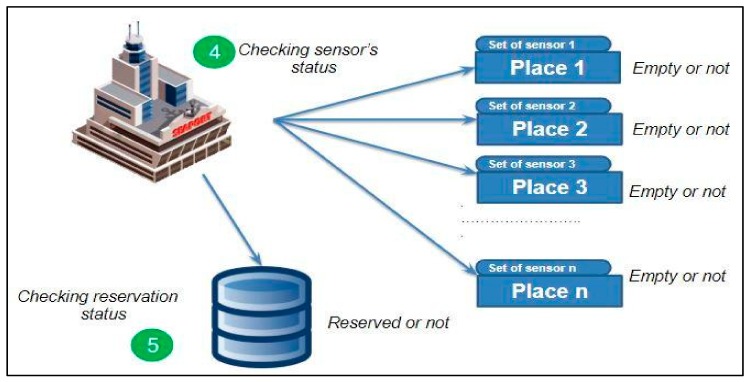
System scenario, steps 4 and 5 [37,55].

**Figure 9:**
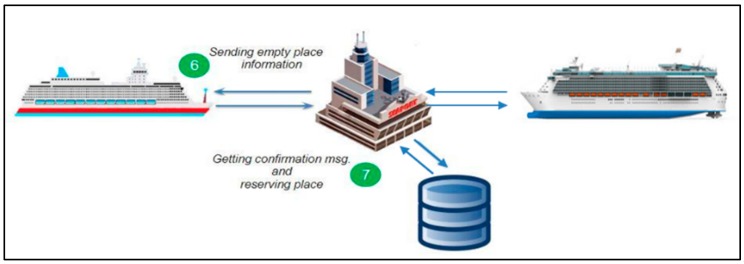
System scenario, steps 6 and 7 [37,55].

So, how does the system work? As we mentioned before, this work is an extended version of our previous work presented in [55]. That is why the proposed system is almost the same as the previous one. The main difference between them is in this current system has multiple sensors at some places (especially bigger places by size) in the port, and in the previous one, we only used one sensor for each place. Its working scenario is as follows:

- Step 1: hips arriving at the port access the port system through the network for instance, using 5G, LTE (LTE-M, LTE-A), Wi-Fi, or WiMAX. When the ship arrives near the port, it sends a connection request to the port.
- Step 2: After receiving a successful response from the port, the ship can send data (information about the ship) and ask for a place to berth.
- Step 3: The port will receive and check the data which is sent from the ship. Figure 7 shows our system scenario from Step 1 to Step 3.
- Step 4: After the port receives the data about the ship, the system at the port will get information about the availability of berthing places using a set of ultrasonic sensors placed in certain locations. The port system sorts places into busy places, empty places, not matched places and reserved places.
- Step 5: After obtaining data on the available vacancies, the system takes into account the reserved places that were requested by ships that arrived at the port earlier and do not match places where the size of the ship does not match the berth. Figure 8 illustrates Step 4 to Step 5 during sorting.
- Step 6: All the collected data concerning the vacancies is sorted and the perfect place location is sent to the ship. The system sends all information about this optimal vacancy such as location and number on a digital map. After sending the place information system it awaits confirmation messages from the ship.
- Step 7: The data regarding the places available for the berthing and the area shown on the port map are sent to the ship. Once a vessel has reserved a place described in the message sent from the port, it will receive a reserved place status. Figure 9 illustrates the system scenario from Step 6 to Step 7.

#### 4.3.1. Proposed System in the Ship

Some of the details of the system in the ship are listed below:

- All static information about the ship is transferred to the system in advance and it is stored constantly (the model, type, color, length, name, owner of the ship, etc.). Moreover, the system keeps all the data about the ports and target places to berth (coordinates, maps, etc.).
- The system receives continuous information with the help of the sensors fixed on the ship (the direction, speed, location, temperature, etc. of the ship).
- The system offers the opportunity for the responsible person (he may be a captain) to add and save the data.

#### 4.3.2. Proposed System at the Port

Some of the details of the system in the port are illustrated below:

- All the static data about possible berthing places and set of sensors is inserted into the system beforehand and it is kept constantly (the measurements of the berths, type of berth, location, number, the ID which is assigned to a set of sensors, etc.).
- The system receives data continuously via the set of ultrasonic sensors that are fixed at different places (is the place vacant or occupied and if it is occupied, it clarifies which ship has been moored there).
- Getting the data taken from a set of sensors the system determines vacant and convenient spacea and it sends the information about the place to a certain ship (the number of the place, location, type of place, etc.).
- The system, before sending the data about vacant places to a certain ship via sensors checks the information of the ships which have reserved the place beforehand and then those places are sorted out from the list. The system sorts berths by the size and type of the ships. Because longer vessels need longer berths and smaller ones need less space. Passenger or cargo ships need particular types of place at the port.

#### 4.3.3. Communication Technologies

The latest 5G, 4th Generation (4G) technologies WiMAX and LTE (LTE-A, LTE-M) are good candidates for the network layer of our project because both have very high transfer rates and very long range for communication. These qualities are very important for communication between ships and ports from a distance. That is why as a network layer for our system we prefer to select one of these two technologies. Some information and a comparison about LTE and WiMAX and their evolution is given in Table 1 below [56].

Worldwide Interoperability for Microwave Access X (WiMAX), is the fourth-generation mobile broadband technology approved by the International Telecommunication Union (ITU), which attempts to mimic the capabilities of Wi-Fi wireless internet, but over a mobile network using an open protocol (802.16 m).

LTE stands for “Long Term Evolution,” and is an ITU-approved 4G mobile broadband technology. It is a direct competitor to WiMAX.

LTE and WiMAX are IP protocols, which makes them better for burst data traffic with good VoIP support. Both use Orthogonal Frequency Division Multiple Access (OFDMA), and multiple access technology which is a form of FDM in which subcarriers are orthogonal to each other.

## 5. Implementation and Experiment

Making the procedure of experiment and implementation in the whole ship and at the port is quite a complicated task. Nevertheless, the experiments of the system purposed for vessels and ports could be carried out in small measure and in small systems. We have done our research as illustrated in Table 2 where the technique and technologies which are important to carry out the experiment are outlined.

**Figure 10:**
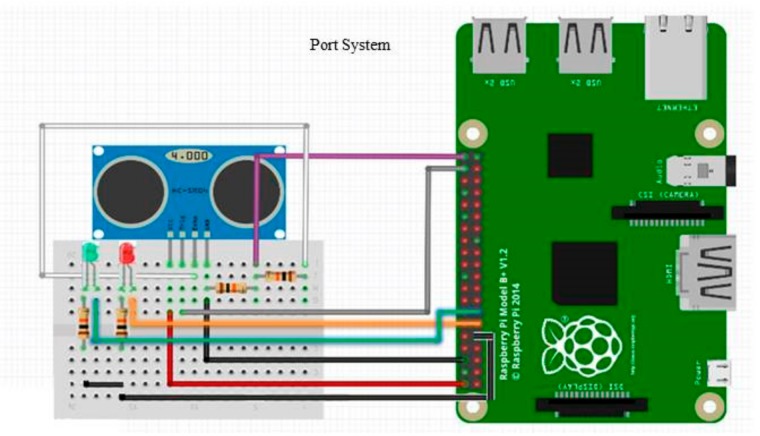
Hardware implementation of the proposed port system [37,55].

In the experimental part, a smartphone and its software that work in Android OC manage the tasks of the vessels. Raspberry Pi and the set of ultrasonic sensors connected to the Raspberry Pi are responsible for transferring data regarding the berths at the port. The system presented is based on the three-layer IoT architecture, where for the application layer we created an application that uses Android Studio via an Android OS smartphone. The dominant language for smartphone application development, Java, is also applied on this layer. In the network layer, we preferred to apply Wi-Fi technology. However, according to the IoT concept, Wi-Fi works as a means of communication between the application layer and the sensor layer. In the sensor layer, the entire intended work in the port system is performed by Raspberry Pi and also by a set of ultrasonic sensors connected to Raspberry Pi. In this part, Noobs Raspbian (Linux OS) is launched on Raspberry Pi. The system is set based on the Python programming language to accept information from the sensor. The server collects information and performs commands via the sensors attached to the Raspberry device. In Figure 10, the experimental hardware implementation for the proposed port system is illustrated. In this work, we add an extra ultrasonic sensor for the berthing places and connect it as the first sensor.

## 6. Results

**Figure 11:**
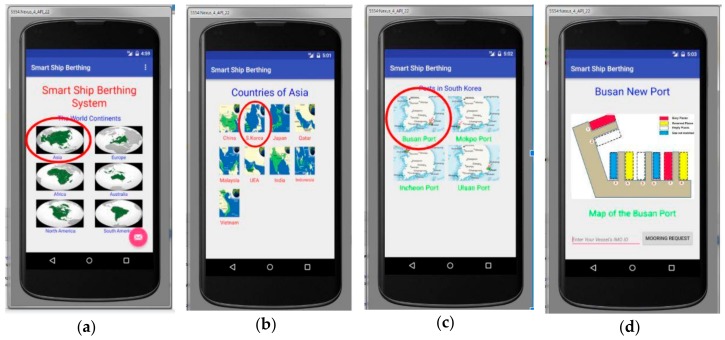
(**a**) Selecting Continent; (**b**) Selecting Country; (**c**) Selecting Port; (**d**) Port Map [37,55].

From this part, we can get an idea of the smartphone application for the proposed ship system. We have created a well-designed application running on Android OC called “Ship Berthing”. There is an underdeveloped business scenario for the application. In order to berth a ship, users need only make a connection with the port system and request a place to berth. To choose a specific port, the user must be notified of the location of the port, for instance country, city, continent, etc. The activities of the application and the scenario are as follows: Run the application, where the first activity page is “Identify the Continent” in which the user has to choose the continent where the next country and port are. We can see this activity in Figure 11a. The second activity page is “Country Selection” where the user must identify the country in which the port the vessels are arriving is located and this activity is shown in Figure 11b. The third page of the activity is the “Port Selection” in which the user must specify the port of arrival as shown in Figure 11c. Finally, “Port Activity” is the fourth main activity page from which the user can send information related to the vessel (in this case, the ship’s IMO ID number) and request a place to berth by clicking the “Request MOORING” button. In addition, users can check the status of this port to see vacancies or occupied berths by the port map on the Internet and this activity is highlighted in Figure 11d. In the online map, the red places indicate the places occupied by ships at the moment. The yellow places indicate that they are also busy, but there are no ships at the moment, which means that these places are reserved for newly arriving ships that will soon berth. Blue places indicate empty berths but not identical places by size or by type. Finally, the white places indicate the empty spaces proportional to size and type which means that the ship can berth in those places.

**Figure 12:**
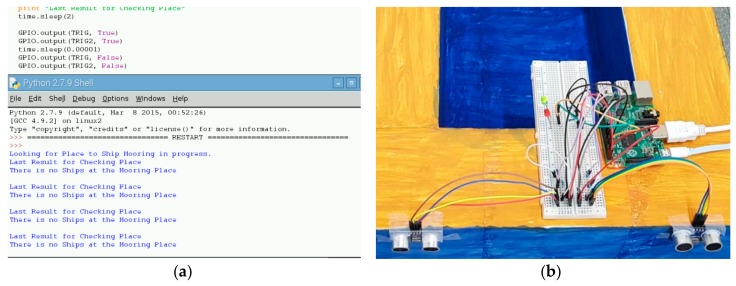

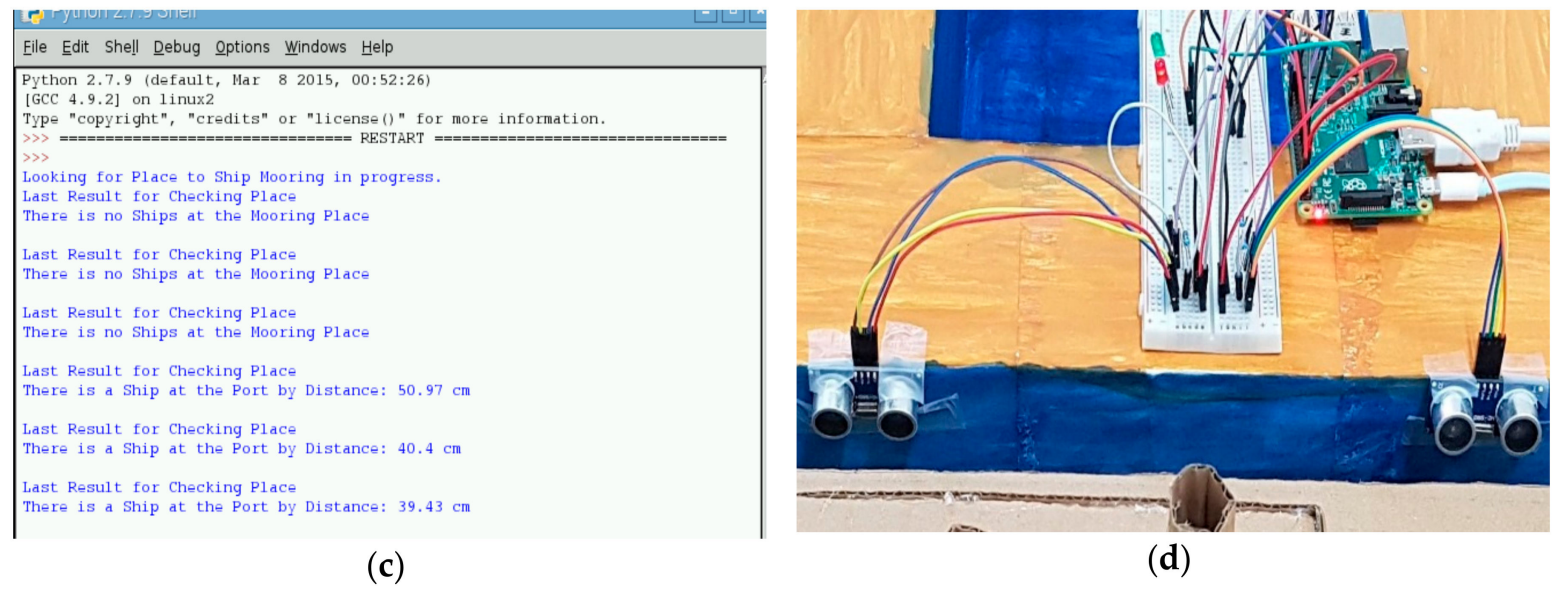
(**a**) Sensor status is empty (Msg result); (**b**) Sensor status is empty (LED result); (**c**) Sensor status is busy (Msg result); (**d**) Sensor status is busy (LED result).

Now we can examine our experimental results for the proposed port system which is devoted to checking vacant places via a set of HC-SR04 UR sensors. This sensor is able to detect an object from approximately 4 m in front and by the detection results the system can determine if there is a ship or not. In the experiment, we used two ultrasonic sensors as a set of sensors for one berthing place to detect and check the status of that place. In order to read sensor information, we utilize Python Language on Raspberry Pi microcontroller which works on Noobs OC. Moreover, we use two, red and green LEDs to set the sensor status as “occupied” or “vacant”. A red LED indicates a sensor which is busy, that means there is a ship at the location. A green LED indicates a vacant sensor that means the place is also empty to moor. Figure 12a,b illustrate the status of the sensor when the berthing place is vacant. Here the green LED is on. Figure 12c,d illustrate the sensor status which the mooring place is occupied. Here, the green LED is on therefore, vessels have to wait to berth.

## 7. Discussion

It is indisputable that a great deal of brand new technologies such as the IoT, big data, and cloud computing are nowadays influencing every aspect in our life. Moreover, in the branch of marine technology, these techniques are also being applied to access more and better services and to automate marine-related operations as well as creating novel smart devices. As a result of this, traditional ports and ships are being substituted by smart ports and vessels. Today the departure and arrival of the ships at port is getting very busy, so ships arriving at a port may not be able to find a place to berth easily which is a complicated situation. Our IoT-based smart berthing system can help vessels and ports in such a situation. This system helps berth the vessels which have arrived at the port and helps exchange data regarding their situation. In the experimental part, a smartphone and its software that works in Android OC manage the tasks of the vessels. Raspberry Pi and a set of ultrasonic sensors connected to the Raspberry Pi are responsible for transferring data regarding the status of berths at the port. The system presented is based on a three-layer IoT architecture. For the application layer we created an application that uses Android Studio via an Android OS smartphone. The dominant language for smartphone application development, Java, is also applied in this layer. In the network layer, we preferred to apply Wi-Fi technology. However, according to the IoT concept, Wi-Fi works as a means of communication between the application layer and the sensor layer. In the sensor layer, the entire intended work in the port system is performed by Raspberry Pi and also by a set of ultrasonic sensors connected to Raspberry Pi. In this part, Noobs Raspbian (Linux OS) is launched on Raspberry Pi. The system is set based on the Python programming language to accept information from the sensor. According to the results of the experiments with our proposed system, sensors and intelligent devices can be used in the marine industry. To test our proposed server-side system, we used a set of ultrasonic sensors to detect a ship and identify a suitable empty place for it to berth. The ultrasonic sensor has a range of about 4 m and a very high resolution and can detect a large object such as a vessel in front of the sensor. This proposed system will be a part of the next level of the maritime industry and will help to develop large projects such as smart ports and smart ships.

## 8. Conclusions

This paper is dedicated to the topic of an IoT-based ship berthing system which utilizes a set of ultrasonic sensors. The system is organized to automate the process of berthing a ship at a port. The main goal of our IoT-based system is achieved by means of a set of sensors installed in the port to automatically identify vacant berths and transfer the corresponding data to ships aiming to dock at the port. The target system is convenient to reduce the time, effort and cost during the berthing process. The purpose of this paper is to provide a dedicated an IoT-based system for automating the procedure by searching for available locations in the via port-mounted sensors and planned ship notification. In the experimental system, we have used smartphone as an alternative to the client-side vessel of the system and created an Android app called “Smart Ship Berthing” instead of a program like NORIVIS 4, VDASH, ODYSSEY, etc. To test our proposed server-side system, we used Raspberry Pi combined with an ultrasonic sensor to detect the ship and identify a vacant berth for it to anchor. The experimental results show that the set of UR sensors has high enough accuracy to detect ships at the port for ship berthing and our proposed system is very amenable to implementation in the real marine environment. In our future work, plan to expand the features of our proposed system especially with an automated ship reporting system which can be an element of our system. Furthermore, we intend to perform studies on big data, and cloud solutions for smart ships and smart ports.

## Figures and Tables

**Table 1:** Comparison of LTE and WiMAX.

Communication Technology	WiMAX	LTE
**Channel Rate**	3.5 to 10 MHz	1.4 to 20 MHz
**Mobility**	Good power efficiency Telecommunication Network (N/w)	Good power efficiency Telecommunication network (n/w)
**Transfer Rate**	4 Mbps downlink 46 Mbps uplink	75 Mbps downlink 300 Mbps uplink
**Coverage**	It supports 60–120 km/h signal speed 50 km range	It supports 350 km/h signal speed 100 km range
**Deployment and Release**	Deployment is good Available in 149 countries, WiMAX 592 network Developed in 2005	Not very widespread Developed in 2009

**Table 2:** Experimental requirements.

Hardware Req.	Software Req.	Platform and Lang.	Others
PC Android Smartphone Raspberry Pi Wi-Fi Router 8 Gb CD-Card	Windows OS Noobs Raspbian (Linux) Android OS & Android Studio Apache	Java & XML Python MySQL	Sensors Jumper wires Resistor LED

## Appendix A. An Example of a Current Ship and Port Reporting Conversation

**Opening:**

OFFICER - Busan Port Control, Busan Port Control. This is the vessel TITANIC. How do you read me? Over.

*BUSAN PORT CONTROL (NPC)*: TITANIC. *This is Busan Port Control. I read you excellent (loud and clear). Switch to channel one - two. Over*.

OFFICER - Switching to Channel - 2. Over.

...after a while...

OFFICER - Busan Port Control. This is TITANIC - AGW5, on channel 1 - 2. I am spelling my name for you: «TITANIC, Tango - India - Tango –Alfa - November - India - Charlie. Call sign: Delta-Alfa - Mike- Kilo - Five. Over

**Message(s):**

*BUSAN PORT CONTROL* - TITANIC. *Understood. What is your ETA Fairway Buoy?*

OFFICER - Busan Port Control. This is TITANIC. My ETA Fairway Buoy is: tomorrow, May the sixth, 09.00 h local time. My maximum draught is 10.7 m and my draught forward is 9.8 m.

*BUSAN PORT CONTROL* - TITANIC. *What is your last port of call and your next port* *of call, please? Over.*

OFFICER - My last port of call was Hamburg, Germany. My next port of call is Amsterdam, Over.

*BUSAN PORT CONTROL - What is your cargo? Over.*

OFFICER - I am a fully cellular container vessel. I have general cargo in containers on board. Total number of containers is 1543. My deadweight tonnage is 28,976 tons.

*BUSAN PORT CONTROL - Have you got any dangerous cargo on board?*

OFFICER: I have 896 tons of dangerous cargo, IMO Class 4.2, in containers on deck,

*BUSAN TORT CONTROL - Understood. Call me again when you are three miles off the Fairway buoy. Stand by on channel 1 - 2 for further instructions regarding berthing instructions*.

**Closing:**

OFFICER - Understood. I shall call you again when three miles off the Fairway Buoy. Standing by on channel 1 - 2. Over and out.

**Taking a Pilot Aboard:**

OFFICER - Busan Port Control. This is TITANIC. How do you read me? Over.

*BUSAN PORT CONTROL* - TITANIC. *This is Busan Port Control. Reading you loud and clear. What is your position? Over.*

TITANIC - I am now in position: bearing 297 degrees, Four miles from the Fairway Buoy. Are there any berthing instructions for me? Over.

*BUSAN PORT CONTROL* – TITANIC. *This is Busan Port Control. Sorry, no berthing prospects for the moment. You should reduce your speed and proceed to the anchorage east of the Fairway Buoy. Rig the pilot ladder on the port side. Stand by on channel 1 - 2 for further instructions. Over.*

TITANIC - Understood. Standing by on channel 1 - 2.

After waiting for two hours at anchor the Master of TITANIC has received the information on the berthing instructions and is rising anchor, waiting for the pilot.

*WATCH OFFICER - There is a pilot launch coming. Master*

OFFICER - Lower the pilot ladder over the port side. Make a lee for the pilot boat.

With the pilot on board the TITANIC proceeds to her berth

*PILOT - What is your heading now?*

OFFICER -3 - 1 - 2, Sir

*PILOT - Very well, keep that course.*

OFFICER - Course 3 - 1 - 2, Sir.

*PILOT - There has been a collision over there. Keep clear of that place. Wait for that big tanker to pass clear ahead of you.*

OFFICER - Very well.

*PILOT - The tide is failing and there is a shoal just ahead of your berth, so you have to be careful while mooring.*

OFFICER - Shall we keep the present course?

*PILOT - I advise you to alter course to 75° when abeam of that buoy.*

OFFICER - Where shall we take the boarding officers?

*PILOT - In the inner road.*
